# Vancomycin Resistance in *Enterococcus* and *Staphylococcus aureus*

**DOI:** 10.3390/microorganisms11010024

**Published:** 2022-12-21

**Authors:** Gen Li, Mark J. Walker, David M. P. De Oliveira

**Affiliations:** School of Chemistry and Molecular Biosciences, Australian Infectious Diseases Research Centre, The University of Queensland, St Lucia, QLD 4072, Australia

**Keywords:** antibiotic, vancomycin, drug-resistance, *Enterococcus*, *Staphylococcus aureus*

## Abstract

*Enterococcus faecalis*, *Enterococcus faecium* and *Staphylococcus aureus* are both common commensals and major opportunistic human pathogens. In recent decades, these bacteria have acquired broad resistance to several major classes of antibiotics, including commonly employed glycopeptides. Exemplified by resistance to vancomycin, glycopeptide resistance is mediated through intrinsic gene mutations, and/or transferrable *van* resistance gene cassette-carrying mobile genetic elements. Here, this review will discuss the epidemiology of vancomycin-resistant *Enterococcus* and *S. aureus* in healthcare, community, and agricultural settings, explore vancomycin resistance in the context of *van* and non-*van* mediated resistance development and provide insights into alternative therapeutic approaches aimed at treating drug-resistant *Enterococcus* and *S. aureus* infections.

## 1. Introduction

### 1.1. Enterococcus faecalis and Enterococcus faecium 

The genus *Enterococcus* are Gram-positive, facultative anaerobic cocci. These bacteria are common commensals of the human gastrointestinal [1] and vaginal tracts, oral cavity [2] and are ubiquitous in nature [3]. In healthy individuals, enterococci can comprise up to 1% of the total bacterial microbiota [4]. Currently, at least 73 different enterococcal species are known [5], with *Enterococcus faecalis* and *E. faecium* being the most common species in humans [4]. Enterococci are also opportunistic human pathogens, with *E. faecalis* and *E. faecium* demonstrating the highest prevalence of infection; up to 90% of human *Enterococcus* infections are caused by *E. faecalis* [6] and the remainder by *E. faecium* [2], although infections by other *Enterococcus* species do sporadically occur [7]. As such, *E. faecalis* and *E. faecium* will be the focus for this review. 

**Figure 1:**
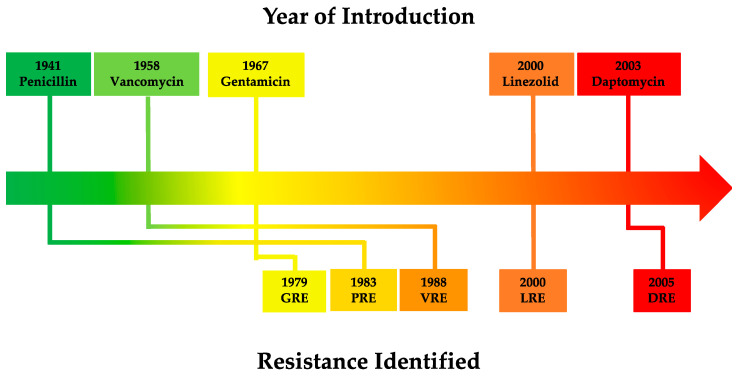
Timeline of antibiotic introduction (above) and subsequent resistance emergence in *Enterococcus* spp (below) [29,30,31,32,33,34]. Abbreviations: GRE—Gentamicin-resistant *Enterococcus*; PRE—Penicillin-resistant *Enterococcus*; VRE—Vancomycin-resistant *Enterococcus*; LRE—Linezolid-resistant *Enterococcus*; DRE—Daptomycin-resistant *Enterococcus*.

Enterococci are intrinsically resistant to many antibiotic classes [8]. Mainly driven by selection pressures caused by inappropriate antibiotic stewardship practices [9], enterococci have acquired additional resistance determinants (Figure 1) through both horizontal gene transfer and spontaneous mutations (Table 1) [10]. *E. faecalis* and *E. faecium* are responsible for numerous nosocomial infections such as wound and soft-tissue infections, neonatal infections, urinary tract infections, meningitis, bacteremia, sepsis, biofilm-associated infections of medical devices and endocarditis [1,11,12]. In humans, *E. faecalis* is the species responsible for the majority of enterococcal infections [6] and has been associated with community-associated (CA) diseases of the oral cavity such as periodontitis, peri-implantitis, caries and endodontic infections [13,14,15] as well as bacteremia, while *E. faecium* is predominantly linked to healthcare-associated (HA) bacteremia [16]. The greater propensity of *E. faecalis* to cause infections can be attributed to its enhanced capability to acquire and express select virulence factors. In contrast, *E. faecium* is considered less virulent but with comparatively higher mortality rates in some HA infections such as bacteremia due to its greater disposition for antibiotic resistance [16,17,18,19,20], including vancomycin resistance [8,21,22,23]. This enhances the survivability and persistence of *E. faecium* within HA settings, allowing it to cause nosocomial infections despite its antibiotic-abundant environment [18,24,25,26,27,28].

**Table 1:** Genetic basis of antibiotic resistance mechanisms in enterococci. There is significant overlap of the numerous different genes and gene mutations implicated in enterococcal and staphylococcal antibiotic resistance (Table 2). Many of these genes are also found on mobile genetic elements (MGEs), which can enable inter- and/or intra-species antibiotic resistance gene transfer [35]. In addition, bacteria can develop/acquire multiple methods of resistance against the same antibiotic class (e.g., mutations in DNA gyrase, topoisomerase IV or the expression of protective proteins or efflux pumps against quinolones). Finally, the expression of one gene may also confer resistance to multiple antibiotic classes (e.g., *cfr*, *optrA*).

Antibiotic Class	Resistance Gene(s), Family or Operon	Protein(s) Produced	Mechanism of Action	Gene Location(s)	Enterococcal Mobile Genetic Elements (MGEs)	References
Aminoglycosides	*aac*	Acetyltransferase	Antibiotic modification and inactivation	Chromosome, plasmid, transposon	Plasmids (P): pIP800, pJH1, pR538-1, pYN134, Inc. 18 Transposons (T): Tn*1546,* Tn*4001*, Tn*5281,* Tn*5382*, Tn*5385*	[36,37,38,39,40,41,42,43,44,45,46,47,48,49,50,51,52,53,54,55]
	*aad,* *ant*	Adenylyltransferase		Plasmid, transposon		
	*aph*	Phosphotransferase		Plasmid, transposon		
	*efmM*	Methyltransferase	Methylation of 16S rRNA nucleotide; reduction of antibiotic target affinity	Chromosome	-	[56]
Bacitracin, cephalosporins	*croRS* system	Penicillin-binding protein 5 (PBP5) and others	Cellular signaling in response to cell wall stress; deletion increases cellular susceptibility to antibiotics. Also involved in overexpression of PBP5	Chromosome	-	[57]
β-lactams	*blaZ*	β-lactamase	Inactivation of β-lactam antibiotics through enzymatic hydrolysis	Chromosome, plasmid, transposon	P: pBEM10 T: Tn*5385*, Tn*552*	[45,46,58,59,60,61,62]
Cephalosporin class β-lactams	*pbp5*	Penicillin-binding protein	Reduced antibiotic affinity; enables cell wall cross-linking in the presence of β-lactams	Chromosome, plasmid, transposon	P: The possibility of plasmid-mediated *pbp5* transfer has been mentioned. No *pbp5*-carrying plasmids have been described in *E. faecalis* or *E. faecium*, although it has been hypothesized.T: Conjugative transposon CTn*5386*	[63,64,65,66,67,68,69]
	*IreK, ireP*	*IreK*—Ser/Thr kinase*ireP*—protein phosphatase	Part of a signaling transduction pathway that regulates cephalosporin resistance	Chromosome	-	[70]
Chloramphenicol	*cat*	Chloramphenicol acetyltransferase	Enzymatic acetylation of chloramphenicol; antibiotic inactivation	Plasmid	P: pRE25, pRUM, pIP501	[71,72,73,74]
Glycopeptides (e.g., vancomycin)	*liaFSR, liaXYZ*	*liaFSR* is a regulatory system, with *liaXYZ* proteins being effector proteins	Modification of the cell membrane and envelope stress response. Modulates cell membrane localization or content, thus altering the antibiotic target site	Chromosome	-	[75,76]
	*cls*	Cardiolipin synthase	Involved in cell membrane synthesis; increased *cls* expression led to membrane modification that impaired antibiotic penetration and activity	Chromosome	-	[77,78,79]
	*mprF*	Bifunctional membrane enzyme involved in phospholipid synthesis and translocation	Increased positive charge of cell membrane and change of membrane fluidity that reduces antibiotic affinity; target modification	Homologs/paralogs described but not extensively studied—gene loci not reported	Unknown	[80,81]
	*van* operon (e.g., *vanA*)	*van* operon proteins—refer to Section 2.3	Reduction of antibiotic affinity through cell wall modification	Chromosome, plasmid, transposon	P: pHKK701, pHKK702, pHK703, pIP816, pIP964, pMG2200, pVEF1 T: Tn*1546*, Tn*1547*, Tn*1549*-like, Tn*5482*, Tn*5506*	[82,83,84,85,86,87,88,89,90,91]
Lincosamides Oxazolidinones Phenicols Pleuromutilins Streptogramin A	*cfr*	rRNA methyltransferase	Methylation of A2503 bacterial 23S rRNA gene; reduced antibiotic affinity to methylated ribosomes	Chromosome, plasmid, transposon	P: pEF-01 T: IS*1216*, Tn*6218*-like	[92,93,94]
	*optrA ^a^*	ABC-F protein	Active dislodgement of antibiotic from its ribosomal target site	Chromosome, plasmid, transposon	P: Inc18, pE349 T: Tn*554*, Tn*6674*	[95,96,97,98,99]
Linezolid	*G2576T*	Point mutation in 23S rRNA gene	Ribosomal target modification, reduction of antibiotic affinity	Chromosome	-	[100]
Macrolides Lincosamides Streptogramins	*erm*	Ribosomal methylase	Methylation of bacterial 23S rRNA domain V; modification of target site and reduced antibiotic binding affinity	Plasmid, transposon	P: pLG2, pRUM-like T: Tn*916*/Tn*1545*	[45,46,58,101,102,103,104,105,106,107,108]
	*lsa*	Efflux pump	Antibiotic efflux	Chromosome, plasmid	P: pMG1-like, pXD4, pY13	[109,110,111]
	*msrA ^b^*			Chromosome	-	[112,113]
Phosphonic Acid (e.g., fosfomycin)	*fosB*	Fosfomycin inactivating enzyme	Mn^2+^-dependent enzymatic modification and inactivation of fosfomycin	Plasmid, transposon, transferable extrachromosomal intermediate	P: pEMA120 T: ISL*3*-like, Tn*1546*-like	[114,115,116,117]
Quinolones	*emeA*	Efflux pump	Antibiotic efflux	Chromosome	-	[118]
	*gyrA*	DNA gyrase mutation	Reduced antibiotic binding affinity			[119,120,121,122]
	*parC*	Mutation of topoisomerase IV				[119,121,122]
	*qnr*	Pentapeptide repeat protein	Protection of DNA gyrase against antibiotic mediated inhibition			[123]
Streptogramin A	*vat*	Acetyltransferases	Antibiotic modification and inactivation	Plasmid	P: pAT15, pAT421	[124,125,126,127,128,129,130]
	*vga*	Efflux pump	Antibiotic efflux	Plasmid *^c^*	-	[130]
Tetracyclines	*tet*M*, tet*O*, tet*S	Ribosome protection protein	Binding to bacterial ribosome; interference with tetracycline-ribosome binding	Chromosome, plasmid, transposon	P: pDO1–like T: Tn*916*/Tn*1545* family, Tn*5397*-like	[45,46,107,108,119,131,132,133,134,135]
	*tet*K*, tet*L	Efflux pump	Antibiotic efflux			

*^a^* Confers resistance to oxazolidinones and phenicols only [99,136]. *^b^* Confers resistance to macrolides and streptogramins only [137]. *^c^* The authors did not designate a name for the plasmid from which the *vga* gene was identified from [130].

**Table 2:** Genetic basis of antibiotic resistance mechanisms in *S. aureus*.

Antibiotic Class	Resistance gene(s), Family or Operon	Protein(s) produced	Mechanism of Action	Gene Location(s) in *S. aureus*	Staphylococcal MGEs	References
Aminoglycosides	*aac*	Acetyltransferase	Antibiotic modification and inactivation	Chromosome, plasmid, transposon	P: pETB_TY825_, pSK41, pUR1902, pUR2941 T: IS*1181*, IS*1182*, Tn*4001*, Tn*5404*, Tn*5405* Tn*554*	[39,40,41,42,43,44,138,139,140,141,142,143,144,145,146,147,148]
	*aad, ant*	Adenylyltransferase				
	*aph*	Phosphotransferase				
β-lactams	*blaZ*	β-lactamase	Inactivation of β-lactam antibiotics through enzymatic hydrolysis	Chromosome, plasmid, transposon	P: pETB_TY825_, pI258, pI9789 T: Tn*552*	[58,59,145,149,150,151,152,153,154,155,156,157,158,159]
Cephalosporins, methicillin	*mecA*	Penicillin-binding protein 2a (PBP2a)	Reduced antibiotic affinity; enables cell wall cross-linking in the presence of β-lactams	Chromosome, pathogenicity island (PAI)	PAI: SCC*mec*	[149,160,161,162,163,164]
Chloramphenicol	*cat*	Chloramphenicol acetyltransferase	Enzymatic acetylation of chloramphenicol; antibiotic inactivation	Plasmid	P: pC194, pC221, pUB112	[165,166,167,168,169,170]
Glycopeptides (e.g., vancomycin)	*cls*	Cardiolipin synthase	Involved in cell membrane synthesis; increased *cls* expression led to membrane modification that impaired antibiotic penetration and activity	Chromosome	-	[171,172,173]
	*mprF*	Bifunctional membrane enzyme involved in phospholipid synthesis and translocation	Increased positive charge of cell membrane and change of membrane fluidity that reduces antibiotic affinity; target modification			[174,175,176]
	*rpoB*	β-subunit of bacterial RNA polymerase	*rpoB* mutations are frequent in vancomycin-intermediate *S. aureus* (VISA) strains. They also lead to upregulation of capsule synthesis, attenuated virulence and immune evasion			[177,178,179]
	*walKR* (also known as *yycFG*)	Inducible two-component regulator system consisting of a sensor kinase and response regulator	Regulation of cell wall synthesis (thickening), biofilm formation, virulence, immune evasion, autolysis			[180,181,182,183]
	*vraS/vraR (vraSR)*		Stress sensing and regulatory system that overproduces protective enzymes such as penicillin-binding protein 2 (PBP2) and other cell wall biosynthesis genes in response to antibiotic activity			[177,184,185,186,187,188]
	*van* operon (e.g., *vanA*)	*van* operon proteins—refer to Section 2.3.	Reduction of antibiotic affinity through cell wall modification	Plasmid, transposon	P: Inc18-like, pLW1043, pSK41-like T: Tn*1546*	[82,189,190,191,192,193]
Fusidic acid	*fusA*	Mutation to the EF-G ribosome complex	Antibiotic target modification; reduced antibiotic affinity	Chromosome	-	[194]
	*fusB*	FusB protein	Prevention of antibiotic interaction with EF-G target site of bacterial ribosome	Chromosome, plasmid, transposon	P: pUB101 T: IS*431/257*	[194,195,196]
	*fusC*	FusC protein		Chromosome, PAI	PAI: SCC_476_, SCC*mec*_N1_, pseudo SCC*mec*-SCC-SCC*_CRISPR_*	[194,197,198,199,200,201]
Lincosamides Oxazolidinones Phenicols Pleuromutilins Streptogramin A	*cfr*	rRNA methyltransferase	Methylation of A2503 bacterial 23S rRNA gene; reduced antibiotic affinity to methylated ribosomes	Chromosome, plasmid, transposon	P: pSCFS3-like, pSCFS7, pSM19035 T: IS*21-558*, Tn*558*	[202,203,204,205,206,207,208,209,210]
	*optrA* ^a^	ABC-F protein	Active dislodgement of antibiotics from the ribosomal target site	Chromosome, transposon	T: Tn*6823*	[95,99,211]
Linezolid	*G2576T*	Point mutation in 23S rRNA gene	Ribosomal target modification, reduction of antibiotic affinity	Chromosome	-	[212]
Macrolides Lincosamides Streptogramins (MLS)	*erm*	Ribosomal methylase	Methylation of bacterial 23S rRNA domain V; modification of target site and reduced antibiotic binding affinity	Chromosome, plasmid, transposon	P: pE194, pUR1902, pUR2940, pUR2941 T: Tn*551*, Tn*554*	[148,213,214,215,216,217]
	*lsa*	Efflux pump	Antibiotic efflux	Chromosome, plasmid, transposon	P: pV7037 T: Tn*560*	[110,218,219,220]
	*mdeA*			Chromosome	-	[110,221]
	*msrA ^b^*			Plasmid	P: pETB_TY825_, pMS97	[145,222]
Mupirocin	*mupA*	Protein modification	Target modification; reduced antibiotic affinity	Chromosome, plasmid, transposon	P: pJ2947, pXU12 T: IS*257*	[223,224,225,226,227]
Phosphonic acid (e.g., Fosfomycin)	*fosB*	Fosfomycin inactivating enzyme	Mn^2+^-dependent enzymatic modification and inactivation of fosfomycin	Chromosome, PAI, plasmid, transposon	PAI: SsPI15305 P: pET28, pIP1842 T: IS*257*-like *^c^*	[117,228,229,230,231,232,233]
Quinolones	*gyrA, gyrB*	DNA gyrase mutation	Reduced antibiotic binding affinity	Chromosome	-	[213,234]
	*parC, parE*	Mutation of topoisomerase IV				[213,234]
	*norA*	Efflux pump	Antibiotic efflux			[235]
	*qnr*	Pentapeptide repeat protein	Protection of DNA gyrase against antibiotic mediated inhibition	Plasmid *^d^*	-	[236]
Streptogramin A	*vat*	Acetyltransferases	Antibiotic modification and inactivation	Chromosomally located conjugative elements, plasmid, transposon	P: pIP524, pIP680, pIP1156, pIP1714 T: Tn*5406*	[126,127,128,129,213,237]
	*vga*	Efflux pump	Antibiotic efflux	Chromosome, plasmid, transposon	P: pSA-7, pVGA, pUR2355, pUR4128, pUR3036, pUR3937 T: Tn*5406*, Tn*5406*-like, Tn*6133*	[144,237,238,239,240]
Sulfonamides	*sulA*	Dihydropteroate synthase	Enzymatic overproduction of *p*-aminobenzoic acid	Chromosome	-	[213]
Tetracyclines	*tet*K, *tet*L	Efflux pump	Antibiotic efflux	Chromosome, plasmid, transposon	P: pT181, pUR1902, pUR2940, pUR2941, pUSA02 T: Tn1545, Tn*5801*-like (Tn*6014*), Tn*916*	[165,193,241,242,243,244,245,246]
	*tet*M, *tet*O, *tet*S *^e^*	Ribosome protection protein	Binding to bacterial ribosome; interference with tetracycline-ribosome binding			
Trimethoprim	*dfrA*	Dihydrofolate reductase	Production of trimethroprim-resistant dihydrofolate reductase	Chromosome, plasmid, transposon	P: pSK1, pSK639 T: IS*257*, Tn*4003*	[245,247,248,249]
	*dfrB*		Reduced antibiotic binding affinity	Chromosome	-	[250,251]

^a^ Confers resistance to oxazolidinones and phenicols only [99,136]. *^b^* Confers resistance to macrolides and streptogramins only [137]. *^c^* The *fosB5* gene was not part of the IS*257*-like transposon but merely surrounded by two copies of it [231]. *^d^* A Nigerian study revealed a very low prevalence of plasmid-mediated *qnr* genes amongst clinical *S. aureus* isolates. No plasmid designations were provided from the study [236]. Quinolone resistance is caused primarily in Gram-negative bacteria through chromosomal mutations [252]. *^e^ tetS* is carried by staphylococci [246] but has not been explicitly found in *S. aureus* in the literature.

Vancomycin-resistant *Enterococcus* (VRE) is a frequent cause of clinical outbreaks worldwide [253,254]. An example of this is the VRE clonal sequence type 796 (ST796) which was first detected in 2011 in Australia, then quickly spread both nationwide and internationally to New Zealand [255] before also causing outbreaks in European hospitals beginning in December 2017 [256]. 

Globally, the prevalence of antibiotic-resistant enterococcal infections remains high and rising in many different countries around the world, with heavy burdens of disease in both developing and developed nations [257,258,259,260,261,262,263,264,265,266,267]. In 2019, *E. faecalis* and *E. faecium* were attributed to 100,000–250,000 fatalities associated with antimicrobial resistance (AMR) [268]. In the United States, VRE constituted 30% of all HA infections in 2017, resulting in approximately 54,500 hospitalizations and 5400 deaths [29]. A 2021 meta-analysis by Shrestha et al. showed the pooled prevalence of VRE in Asia to be 8.1%, higher than those reported from Europe [269] but lower than North America (21%) [260]. In 2020, the reported overall pooled prevalence of VRE in Africa was 26.8% [270], while Australia had an overall vancomycin resistance rate in *E. faecium* of 32.6% [271], with VRE constituting up to 64.2% of all bloodstream infections in some regions of the country that same year [272]. The overall prevalence of VRE in clinical enterococcal isolates in the South American nations of Columbia, Ecuador, Peru and Venezuela was 31% overall between 2006–2008 [273]. As such, VRE has been designated a “high priority” and “serious threat” pathogen by the World Health Organisation (WHO) [274] and the U.S. Centers for Disease Control and Prevention (CDC) [29], respectively. 

The contrasting geographic burden of disease imposed by VRE across select countries has been shown to typically correlate with national antimicrobial stewardship and surveillance practices. In developed European nations with robust stewardship and surveillance programs [275,276], the prevalence of VRE is much lower than in other developed countries with comparatively modest levels of stewardship such as Australia [277,278]. The observation that lower and middle-income countries in Africa, Asia and South America can have comparable or lower prevalence of VRE to some developed nations such as Australia, despite their absence of quality stewardship and surveillance programs however can be explained by the lack of published epidemiological data from these regions [279,280,281,282,283,284,285]. Therefore, it is likely that the true burden of VRE in Africa, Asia and South America are much higher than the available figures provided from those countries. This assumption is consistent with the results of a 2022 study which showed that the overall burden of AMR in 2019 was highest in sub-Saharan Africa and higher amongst low- and middle-income countries than more developed nations in Australasia, Western Europe, and East Asia [268]. 

The global burden of VRE in food of animal origin was estimated to be 11.7% by Lawpidet et al., in 2021, thought to be driven by the use of avoparcin (a vancomycin analog) within livestock feed for growth promotion. Using meta-analysis, they reported the prevalence of VRE in animal foods to be: Africa (18.5%), Europe (12%), Asia (11.7%), South America (3%) and North America (0.3%). The finding that the frequency of VRE in European animal products was higher than Asia was surprising, and may be explained by the discrepancy in data availability in Asia, the types of studies included in the meta-analysis [286] as well the poor availability of antimicrobial consumption and AMR surveillance data in lower-income countries [287,288]. In addition, the prevalence levels of VRE in healthcare do not always correlate with levels observed in agriculture; in the United States, the prevalence of VRE in HA infections was 30% in 2017 [29], far exceeding the 0.3% figure in North American farms. This may be attributed to the fact that avoparcin was never approved for use in North America [286]. 

Given current trends, it is predicted that antimicrobial consumption—and by extension, prevalence of HA and agricultural VRE—will significantly increase in Africa, Asia (particularly South and Southeast Asia) and South America [289]. Although all continents are predicted to increase their future antimicrobial consumption [290], increases are expected to disproportionally affect developing regions due to their rapid growth, and potential lack of appropriate infection control and stewardship practices [290,291,292,293]. Therefore, future initiatives aimed at reducing antimicrobial use and enhancing antimicrobial stewardship, particularly in developing nations, will need to be balanced with the necessity to provide food security to these low- and middle-income countries [289]. 

### 1.2. Staphylococcus aureus

*Staphylococcus aureus* is a Gram-positive, facultative anaerobic bacterium [294]. Both a commensal as well as a significant pathogen of humans [295], *S. aureus* is prevalent in community, healthcare [296] and agricultural settings [297,298], asymptomatically colonising up to 30% of the human population [299]. 

As one of the most versatile and successful opportunistic human pathogens [296,300], *S. aureus* possesses a large variety of virulence factors [301] that enable host colonisation, tissue damage, immune evasion and progression of disease [301,302]. Consequently, *S. aureus* infections can be grouped into three general categories: (i) toxinoses such as scalded skin syndrome, food poisoning and toxic shock syndrome; (ii) benign and self-limiting conditions such as superficial skin and soft tissue infections; and (iii) systemic, life-threatening complications such as brain abscesses, meningitis, pneumonia, osteomyelitis, endocarditis, bacteremia, multi-organ failure and sepsis which carry high rates of morbidity and mortality [303,304]. 

*S. aureus* infections can be either HA or CA. The characteristics and virulence profiles of CA *S. aureus* typically differ to those of HA *S. aureus* [305,306]. HA infections of methicillin-resistant *S. aureus* (MRSA) were first reported in the 1960s [307], but rarely affected non-hospitalised healthy people and failed to spread efficiently within the community. This was generally attributed to the fitness cost imposed upon HA-MRSA through acquisition of antibiotic resistance elements [308], and is consistent with studies that reported HA-MRSA being generally more drug-resistant [305,309] and have reduced fitness and virulence [310] than CA-MRSA. Nevertheless, HA-MRSA clones remain a major cause of nosocomial infections globally [307].

The global emergence of CA-MRSA [311,312,313,314,315,316] began in the late 1980s [308], and was defined as a MRSA infection in the community whereby the infected persons exhibits no apparent nosocomial risk factors. This suggested that CA-MRSA evolved independently from lineages present in clinical settings. This hypothesis was further supported by the observation that CA-MRSA and HA-MRSA are epidemiologically, clinically, and microbiologically distinct [306,309]. Typically, CA-MRSA differ from HA-MRSA through the former exhibiting low-level susceptibility to non-β-lactam antibiotics, carriage of SCC*mec* types IV or V and production of Panton-Valentine leukocidins [308]. However, possible transmission between HA-MRSA to CA-MRSA may increase the overlap in similarities between the two MRSA sub-populations [306]. 

For HA-MRSA, ST239 was traditionally considered to be the dominant global hospital clone [317] and remains prevalent in Asia along with ST5 [318,319]. Elsewhere, the prevalence distribution of HA-MRSA clones will vary depending on geographical location: USA100 (North America) [320,321,322,323], CC5 (Latin America) [324,325], ST22 (United Kingdom), ST225 (central Europe) [319], ST22-IV [2B] (Australia) [326] and ST5 and ST239/241 (Africa) [327].

In the community, the dominant CA-MRSA clone also varies by geographical location: USA300—ST8-IV (North America), USA1100 and USA300-Latin American variant (South America), ST80-IV (Europe), ST93-IV (Australia), high heterogeneity in Asia (no dominant clone) and insufficient data for Africa [328]. Although traditionally considered a HA pathogen, the burden of CA-MRSA disease has been on the rise since its global emergence in the 1990s [329] and it began to appear within HA facilities in the 2000s [308]. Since then, many countries such as Australia [330], China [319], India [331], Kuwait [332], South Korea [333,334] Switzerland [335] United Arab Emirates [336], United Kingdom [337] and the United States [338,339,340] have reported the occurrences of persistence, dissemination, outbreaks and/or outright dominance of CA-MRSA clones within HA facilities which are attributed to the comparatively higher fitness of CA-MRSA through its carriage of smaller SCC*mec* variants and fewer, if any, other antibiotic resistance determinants [310].

The exact mechanisms driving the divergent evolution of MRSA clones, and reasons for the emergence and replacement or dominance of specific clones in different geographical locations remain unclear [307,311,341,342,343,344]. We hypothesise that factors such as the host population demographics, migration, environmental climate, presence of other microorganism communities (e.g., other bacteria and bacteriophages that can facilitate horizontal gene transfer), spontaneous gene mutations and level of antibiotic use and stewardship are all likely to play contributing roles. With such changing diversity in MRSA clones, rapid and accurate clinical diagnosis, combined with a tailored treatment regimen according to the resistance profile of the clonal type will be essential for effective patient care [328].

**Figure 2:**
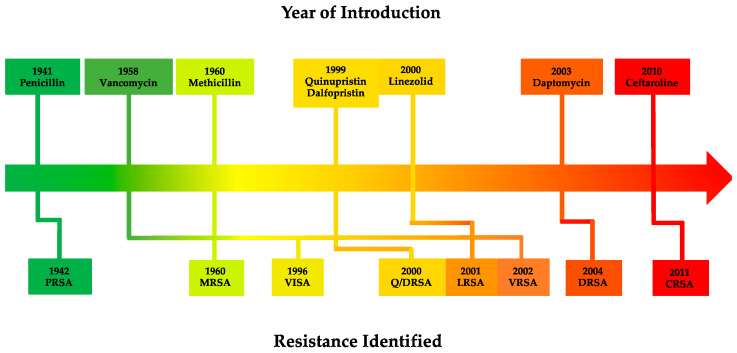
Timeline of antibiotic introduction (above) and subsequent resistance emergence in *S. aureus* (below) [29,30,33,34,345,346]. Abbreviations: PRSA—Penicillin-resistant *S. aureus*; MRSA—Methicillin-resistant *S. aureus*; VISA—Vancomycin-intermediate *S. aureus*; Q/DRSA—Quinupristin/dalfopristin-resistant *S. aureus*; LRSA—Linezolid-resistant *S. aureus*; VRSA—Vancomycin-resistant *S. aureus*; DRSA—Daptomycin-resistant *S. aureus*; CRSA—Ceftaroline-resistant *S. aureus*.

*S. aureus* has demonstrated a remarkable ability to rapidly acquire and develop antibiotic resistance (Figure 2), often achieved through horizontal gene transfer of mobile genetic elements (MGEs) and chromosomal mutations [343]. As a result, an extensive arsenal of resistance mechanisms has emerged in *S. aureus* that enables resistance to major antibiotic classes typically employed to treat infection (Table 2).

Like enterococci, the rapid emergence of resistance development in *S. aureus* has been attributed to the misuse and overuse of antibiotics in clinical and agricultural settings [9]. When combined with additional factors such as high rates of asymptomatic colonisation [299,347] and increased accessibility of international travel, *S. aureus* infections, particularly those caused by antibiotic resistant strains, have reached epidemic proportions in community and clinical settings worldwide [348]. Globally, *S. aureus* was responsible for more than 250,000 deaths associated with AMR in 2019 [268]. In the United States, *S. aureus* caused more than 119,000 bloodstream infections which led to nearly 20,000 deaths in 2017 [349]. As with VRE, antibiotic resistant *S. aureus* has also been designed as a “high priority” and “serious threat” pathogen by the WHO [274] and U.S. CDC [29] respectively. 

## 2. Vancomycin

### 2.1. Discovery and History

Vancomycin is a tricyclic glycopeptide antibiotic first isolated in 1957 from the fungus *Streptomyces orientalis*. *In vitro* experiments showed that it had broad spectrum activity against Gram-positive bacteria, with no detected resistance in staphylococci following serial passages in media containing vancomycin. After showing promising efficacy and safety profiles in animal models, vancomycin (name derived from “vanquish”) entered human clinical trials [33,350]. During an initial clinical trial, vancomycin successfully treated 8 out of 9 patients with severe staphylococcal infection. Therapy failure occurred in one patient who was suffering from empyema, which prevented a therapeutic dose level of vancomycin from being administered [351]. In another human study, 5 out of 6 endocarditis patients who had already experienced antibiotic failure demonstrated resolution of disease indicators; the singular patient who experienced therapy failure had also presented with multiple conditions such as intractable heart failure and shock [352]. 

The culmination of positive data from these respective clinical trials subsequently resulted in the immediate approval of vancomycin by the U.S. Food and Drug Administration (FDA) in 1958. However, due to perceived nephrotoxicity [33,353,354], vancomycin was originally categorized as a last resort medication reserved for patients who were infected with bacteria that were resistant to frontline drugs or those patients with serious allergies to standard therapy [33]. Today, vancomycin is used as a first-line treatment for MRSA [355,356,357], and remains an important antibiotic used against serious Gram-positive bacterial infections [358,359]. 

### 2.2. Mechanism of Action

**Figure 3:**
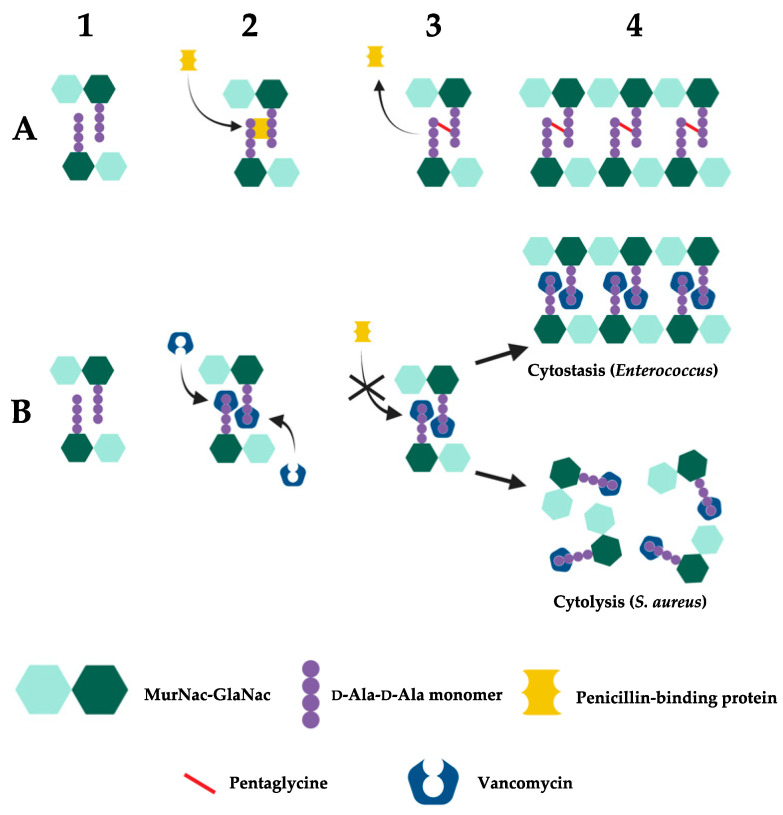
Mechanism of vancomycin activity. (**A**) Susceptible bacteria undergo normal cell wall synthesis through enzymatic (transglycosylase and transpeptidase) cross-linking activity in the absence of vancomycin. 1. Bacterial peptidoglycan with unlinked D-Ala-D-Ala monomers. 2. PBP recognises and binds to D-Ala-D-Ala monomers. 3. PBP facilitates the cross-linking of peptidoglycan D-Ala-D-Ala monomers through catalysis of pentaglycine bonds [23,350,360,361,362,363,364]. 4. Newly formed cell wall with complete cross-linking of D-Ala-D-Ala monomers. (**B**) Vancomycin inhibits peptidoglycan cross-linking in susceptible bacteria through its recognition and binding to D-Ala-D-Ala monomers. 1. Bacterial peptidoglycan with unlinked D-Ala-D-Ala monomers. 2. Vancomycin recognises and binds to D-Ala-D-Ala monomers. 3. Prevention of PBP-mediated catalysis of pentaglycine bonds due to vancomycin’s binding to D-Ala-D-Ala monomers. 4. Peptidoglycan cross-linking is inhibited, disrupting cell wall synthesis which leads to cytostasis (*Enterococcus*) or cell death (*S. aureus*) [23,350,360,361,362,363,364]. Created with BioRender.com.

Vancomycin inhibits the cell wall synthesis of Gram-positive bacteria by binding to D-Ala-D-Ala dipeptide subunits of peptidoglycan monomers anchored to the sugar backbone of alternating N-acetylmuramic acid (MurNac) and N-acetylglucosamine (GlcNac) residues [360,361]. In susceptible bacteria, peptidoglycan monomers normally undergo transglycosylation and transpeptidation by the glycosyltransferase and transpeptidase activities of penicillin-binding proteins (PBPs), forming new peptidoglycan structures through pentaglycine cross-linkage [362,363]. Vancomycin, as a largely hydrophilic molecule, disrupts this process by forming hydrogen bonds to the D-Ala-D-Ala moiety through its aglycon subunit. As a result, this complex leads to a conformational change to the peptidoglycan which prevents subsequent transglycosylase and transpeptidase activity. Consequently, cell wall synthesis is inhibited as new peptidoglycan monomers are unable to be incorporated into the growing peptidoglycan skeleton, eventually leading to bacteriostasis in enterococci [350] or osmotic shock, cell lysis and death in *S. aureus* (Figure 3) [23,350,364,365].

Although adverse effects are still observed from prolonged administration or high concentrations of use, vancomycin’s toxicity has been significantly reduced since its first introduction. This was most likely achieved due to the removal of impurities present in early batches [350]. The improvement in vancomycin’s safety profile, in addition to the emergence of methicillin-resistant bacteria in the 1970s, subsequently lead to its mainstream adoption and use [34]. Today, vancomycin’s utility and importance in modern medicine is highlighted by its inclusion on the WHO’s model list of essential medicines [366]. 

### 2.3. Vancomycin Resistance in Enterococcus

The widespread use of vancomycin has predictably resulted in the rapid emergence and spread of vancomycin resistance amongst various Gram-positive bacteria [350]. In 1988, Uttley and colleagues published the first clinical outbreak of highly resistant VRE, with some isolates having minimum inhibitory concentrations (MICs) greater than 2000 µg/mL [367]. Today, the Clinical Laboratory Standards Institute (CLSI) classifies complete vancomycin resistance in *Enterococcus* with a MIC of ≥32 µg/mL, intermediate resistance as 8–16 µg/mL and susceptible as ≤4 µg/mL using broth microdilution testing [368]. This is consistent with the breakpoints set by the European Committee on Antimicrobial Susceptibility Testing (EUCAST) which define the MIC of vancomycin susceptible enterococci to be ≤4 µg/mL and vancomycin-resistant enterococci to be>4 µg/mL [369]. 

**Figure 4:**
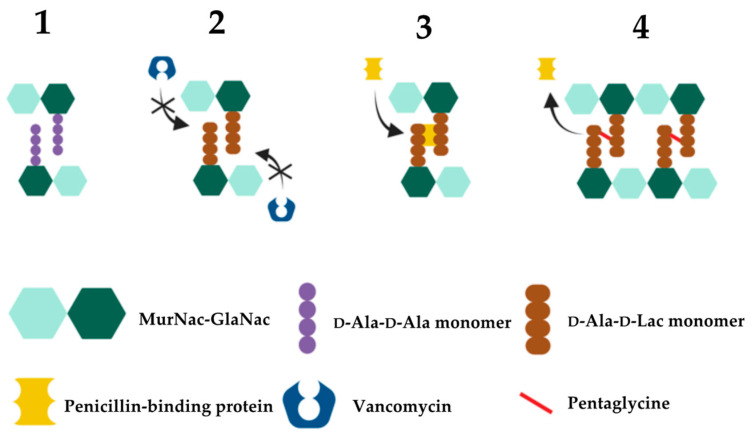
Mechanism of high-level vancomycin resistance. 1. Vancomycin-sensitive bacteria expressing D-Ala-D-Ala monomers. 2. Mutated vancomycin-resistant bacteria expressing D-Ala-D-Lac monomers that vancomycin poorly recognises. 3. With D-Ala-D-Lac not bound to vancomycin, PBP can subsequently bind to and catalyse the formation of pentaglycine bonds between MurNac-GlaNac monomers. 4. Bacterial peptidoglycan cross-linking and cell division continue uninhibited.

Vancomycin resistance in enterococci is centered around the modification of the vancomycin target site i.e., modification of the D-Ala-D-Ala terminal amino acids of dipeptide monomer subunits into either D-Ala-D-Lac or D-Ala-D-Ser (Figure 4). These mutations confer high- and low-level vancomycin resistant phenotypes respectively. This is because the binding affinity of vancomycin for D-Ala-D-Lac is reduced 1000-fold due to loss of a single hydrogen bond [370] compared to its modestly (6-fold) reduced affinity for D-Ala-D-Ser due to steric hindrance by the D-Ser hydroxyl group [371,372]. As the mechanism of resistance (D-Ala-D-Lac or D-Ala-D-Ser) is determined by different *van* cassettes, the degree of vancomycin resistance in enterococci will be dependent upon which *van* operon they express [371]. The different *van* operons, their respective genes, proteins, and mechanisms of action responsible for these variable resistance levels are summarised in Table 3.

Low-level vancomycin resistance mediated by D-Ala-D-Ser monomers follows the same principle as shown here [370,371,372]. Created with BioRender.com. 

**Figure 5:**
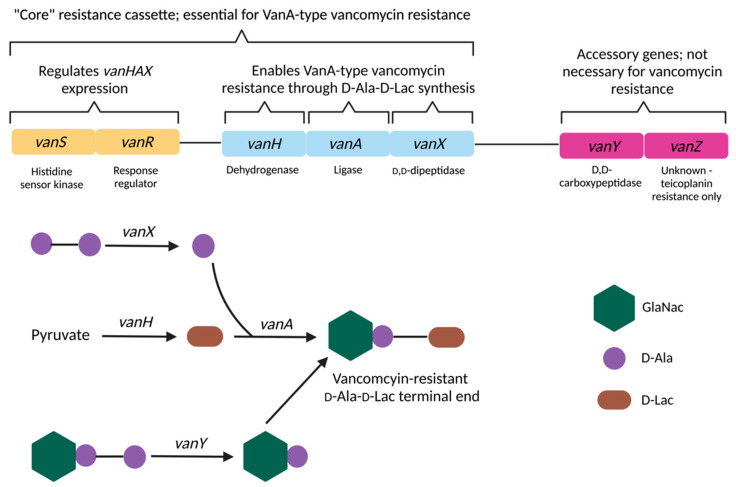
Molecular mechanisms of *vanA*-mediated vancomycin resistance. Expression of *vanHAX* genes are regulated by the two-component *vanSR* system. D-Ala-D-Ala components are hydrolysed by VanX, with VanY hydrolysing terminal D-Ala-D-Ala residues of existing peptidoglycan precursors not eliminated by VanX. Pyruvate is reduced to D-Lac by VanH, and VanA catalyses the esterification of D-Ala to D-Lac to form vancomycin-resistant peptidoglycan terminal ends. The role of VanZ in mediating vancomycin resistance is unknown and is implicated in teicoplanin resistance only [304,399,400]. Created with BioRender.com.

High level, D-Ala-D-Lac based vancomycin resistance is encoded by the *vanA* operon and its homologs *vanB*, *vanD*, *vanF* and *vanM*. The *vanA*, *vanH*, *vanX*, *vanS* and *vanR* genes collectively compose the “core” resistance cassette known as “VanA-type” vancomycin resistance (Figure 5). v*anY* and *vanZ* are considered “accessory” genes of the *vanA* cassette and are not strictly necessary for conferring resistance (Table 3). The naming of homologous genes in all VanA-type operons are identical to each other, with the exception of the ligase gene which is named after its operon i.e., the genes *vanA*, *vanB*, *vanD*, *vanF* and *vanM* encode for the ligase proteins in the vanA, vanB, vanD, vanF and vanM operons but the dehydrogenase gene in all five VanA-type resistance operons are named *vanH* [371]. The *vanA* genotype is the most common amongst VRE and vancomycin-resistant *S. aureus* (VRSA) worldwide [397,398].

The D-Ala-D-Ser type resistance encoded by the *vanC* cassette was discovered in the chromosomes of *Enterococcus gallinarum*, *Enterococcus casseliflavus* and *Enterococcus flavescens*, providing intrinsic, low-level vancomycin resistance [82,397,401,402,403,404,405,406]. Homologs of *vanC*; *vanE*, *vanG*, *vanL* and *vanN* were later found in *E. faecalis* [384,385,407,408], and these operons allow for production of D-Ala-D-Ser peptidoglycan terminals. These cassettes also contain similar genes to those of the VanA-resistance type in addition to two genes exclusive to the D-Ala-D-Ser resistance cassette: a *vanT*-encoded serine racemase and *vanXY*-encoded bifunctional dipeptidase/pentapeptidase (Table 3). The naming of homologous genes in VanC-type resistance cassettes follow the same nomenclature as VanA-type resistance operons [371].

The *vanA*, *vanB*, *vanG*, *vanM* and *vanN* operons are transferable between bacteria. The distribution of these *van* operons in enterococci has been reviewed by Ahmed and Baptiste [397]. For a detailed review on the *van* operons and genes involved in vancomycin resistance, refer to Stogios and Savchenko [371]. 

Although the acquisition of vancomycin resistance via MGEs has been shown to incur a high and immediate fitness cost in enterococci [409], the prevalence of VRE has continually increased globally since emergence in the 1980s [260,270,410,411]. However, even in the absence of vancomycin selection, *van*-carrying enterococcal MGEs have demonstrated high rates of intra-species conjugation, stability within the host [412], impose little to no fitness cost when uninduced [413,414] and rapidly mitigate biological costs upon growth and form beneficial host-plasmid associations [409]. Worryingly, this suggests that antibiotic stewardship and decreased use would be insufficient to reduce the prevalence of VRE in healthcare and community settings. 

This is because of the known *van* operons, the most predominant (*vanA*, followed by *vanB*) are associated with MGEs [415,416] that can carry other multidrug-resistance elements [417]. Therefore, the clinical and community use of non-glycopeptide antibiotics can also co-select for vancomycin resistance in the absence of vancomycin therapy [418]. Antimicrobials are also used in enormous quantities within animal feed in agriculture for growth promotion and disease prevention in livestock [289]. This subsequently places additional resistance selection pressures on the commensal bacteria carried by animals as well as bacteria in the surrounding environment through waste dissemination [288,419]. The presence of environmental heavy metals and use of biocides in agriculture [420,421,422] could also expedite this process.

While reducing inappropriate and excessive antibiotic use through implementation of appropriate stewardship reforms has shown to deliver positive outcomes [278,423,424,425], in practice, antibiotic stewardship can be complex and difficult to carry out [426] due to a multitude of factors such as lack of available information systems, funding, staffing, resourcing, or competition from higher priority initiatives [427]. Depending on the type of stewardship program applied, it on occasion can lead to delayed diagnoses and reduced patient outcome [428].

Even with the appropriate stewardship practices however, there is evidence from the poultry industry that it would only reduce, not eliminate the burden of VRE in agricultural settings. In 1997, the use of avoparcin in farming was banned by the European Union. In 2019, a study by Simm et al. demonstrated that significant reductions in VRE in broilers can be achieved through abolishment of antimicrobials in animal feed in addition to stringent disinfection and cleaning practices [429]. Similar observations of a reduction in VRE burden were observed in other countries following the ban of avoparcin. However, all these measures failed to achieve complete elimination of VRE [430,431,432,433,434]. 

Several theories surrounding the persistence of VRE have been suggested, such as vancomycin resistance co-selection by other antibiotics [397,435] and heavy metals, as well as plasmid addiction systems that force bacteria to retain *vanA*-carrying MGEs [434]. Alongside these factors, we hypothesise that other reasons such as the commensal nature of *Enterococcus* within humans and animals [436,437], its ubiquitous presence in the natural environment [3] and the adaptability, stability and transferability of *van*-containing MGEs [438] also play contributing roles. From a human perspective, these studies suggest that antibiotic stewardship initiatives to reduce glycopeptide use in hospitals and communities would significantly reduce the burden of VRE in endemic areas but may make little difference in completely eliminating VRE in settings with already low VRE prevalence. 

Prolonged vancomycin therapy can lead to the emergence of vancomycin-dependent *E. faecium* [439] and *E. faecalis* (VDE) [440], which was first reported in 1994 [441], whereby bacteria lose or inactivate their functional D-Ala-D-Ala production pathway and become reliant on the presence of vancomycin to stimulate *van*-mediated peptidoglycan synthesis [440,442]. Unfortunately, suspension of vancomycin treatment may be insufficient to cure VDE infections due to the rapid reversion of VDE to vancomycin-independent colonies *in vitro* [440], likely through further mutations that allow re-activation of D-Ala-D-Ala synthesis or constitutive activation of an alternative *van* pathway [82]. Although rare, VDE infections in human patients have been reported in the literature [439,440,441,443,444,445,446,447,448]. However, due to the infrequent nature of such infections, VDE are poorly studied and understood, while optimum treatment guidelines remain unclear [445].

More recently, *vanA*- or *vanB*-carrying enterococci [449] which appear vancomycin-susceptible in traditional phenotypic susceptibility tests [450], but can revert to a vancomycin-resistant phenotype upon vancomycin treatment [451,452] were reported for the first time in 2011 [453]. The ability of these strains, termed vancomycin-variable enterococci (VVE), to evade diagnostic tests and switch phenotypes during antibiotic therapy [452] is thought to be due to major deletions in the Tn*1546 vanA* operon [453] and/or inducible or constitutive *vanHAX* expression [454] which significantly compromises treatment success [452].

Compared to VRE, VVE infections are rare, although several outbreaks have been reported in Europe [455] and North America. To date, limited data is available for the overall prevalence of VVE [454], which may be attributed to the pathogens capacity to evade drug sensitivity screening tests [452]. Therefore, it is likely that current epidemiological estimates of VVE, particularly in developing countries, would be below its actual prevalence value. To combat this, molecular-based testing methods such as PCR are required [450], but the facilities and apparatus for these techniques may be limited in low- and middle-income regions. 

### 2.4. Vancomycin Resistance in S. aureus

In 1996, MRSA which were clinically refractory to vancomycin treatment was reported for the first time in Japan [345]. These strains, named Mu3 and Mu50, had MICs of 3 µg/mL and 8 µg/mL respectively [456] and were later termed hetero-vancomycin-intermediate *S. aureus* (hVISA) and vancomycin-intermediate *S. aureus* (VISA) respectively [457]. In 2002, complete VRSA was reported for the first time in the United States [458]. Today, CLSI defines *S. aureus* complete vancomycin resistance with a MIC of ≥16 µg/mL, intermediate resistance as 4–8 µg/mL and susceptible as ≤2 µg/mL with broth microdilution testing [368]. This is consistent with the breakpoints set by EUCAST which define the MIC of vancomycin susceptible *S. aureus* to be ≤2 µg/mL and vancomycin-resistant *S. aureus* to be >2µg/mL [369]. 

The conversion of vancomycin-susceptible *S. aureus* (VSSA) to VISA occurs through spontaneous mutations in genes such as *walkR*, *rpoB*, *vraSR* and *mprF*. Mutations in these genes are thought to confer the wide range of favorable phenotypic changes in VISA that allow for greater vancomycin resistance such as membrane charge modification, upregulation of cell wall biosynthesis genes, cell wall thickening, biofilm formation and modulation of key cellular processes such as immune evasion, virulence attenuation and reduced autolysis (Table 2) [174,175,176,177,178,179,180,181,182,183,184,185,186,187,188,457].

hVISA is defined as the precursor to VISA, and in most cases emerges as a semi-resistant subpopulation of daughter cells from a previously susceptible but heterogenous VSSA population [457]. Following prolonged vancomycin exposure and selection pressure that favors their outgrowth, eventually a homogenous VISA population is achieved [459]. Interestingly, hVISA may also give rise to “slow” VISA (sVISA), a slower growing VISA subpopulation with similar phenotypes to extant “wild type” VISA but with longer doubling times, higher vancomycin MICs (≥6 µg/mL) and VISA phenotype instability (i.e., rapid reversion to hVISA in the absence of drug selection pressure) [457].

Despite the greater therapeutic difficulty in treating VISA infections, VISA appears to be less virulent than VSSA but with a greater ability to colonise and evade the host immune system [460,461]. Prior studies employing a mouse model of skin and soft tissue infection demonstrated that VISA had a much lower invasive capacity than VSSA; VISA also induced lower levels of innate immunity in persistent and chronic infections [461]. Proposed mechanisms for VISA’s virulence reduction include loss-of-activity mutations or dysfunction of the quorum-sensing, virulence regulator system *agr* [461]. Virulence factors under *agr* control include expression of the α-hemolysin encoded by *hla* [462], with mutant or dysfunctional *agr* VISA isolates found to produce up to 20-fold less α-toxin than VSSA and also less lethal in an *in vivo* murine bacteraemia model [460]. In addition, VISA had a comparatively higher capacity for biofilm formation than VISA, which are key contributors to *S. aureus* immune evasion and persistence. This was supported by the upregulation *fnbB* and *sdrCDE* genes which are associated with cell adhesion and immune evasion respectively [461]. 

However, there does appear to be a fitness cost with VISA phenotypes compared to VSSA [463,464]. The rapid rate of conversion between hVISA and sVISA also demonstrates the high adaptability of *S. aureus*; it can resist vancomycin treatment in the sVISA form and revert to hVISA after treatment to reinstate the infection [457]. Ultimately, the sacrifice of acute virulence for greater antibiotic tolerance and immune evasion in VISA allows for higher host tolerance of the bacteria. Clinically, this manifests as chronic *S. aureus* infections that persist despite recurring rounds of treatment [460,461]. 

The global epidemiology of sVISA has not been well studied, perhaps due to its relatively recent discovery and instability of its phenotype [464]. Contrastingly, one study by Katayama et al. detected sVISA prevalence at 15.6% amongst clinical MRSA isolates, with VISA at less than 1%. This suggests that the known rates of sVISA are likely underestimates of the true figure due to lack of testing [465]. The global burden of VISA and hVISA have been increasing in recent years, particularly in the American and Asian continents. In 2020, Shariati et al. reported the overall global prevalence of VISA and hVISA to be 1.2% and 4% amongst *S. aureus* isolates pre-2010 respectively, which rose 3.6- and 1.3-fold to 4.3% and 5.3% after 2010–2019 respectively. By continent, VISA was most common in Asia (2.1%), followed by Africa and Europe (1.8%), North and South America (1%) and Oceania (0.6%); hVISA was most frequent in Oceania (11.2%), followed by North and South America (5.2%), Asia (4.7%), Europe (4.4%) and Africa (4%) [466]. 

High level vancomycin resistance in *S. aureus* was first reported in 2002 [458] and was acquired through transmission of the *vanA*-containing transposon Tn*1546* from *E. faecalis* plasmids [467], with an identical mechanism of resistance as previously described in Section 2.3 [304]. However, VRSA infections remain rare due to the fitness cost imposed [468] as well as other factors such as limited *vanA* transmission within *S. aureus*, instability of the Tn*1546* transposon-carrying plasmid in *S. aureus* and good antibiotic stewardship [355]. In contrast, because only stepwise mutations are required for VISA conversion [469,470], VISA infections have a comparatively higher burden of disease [466]. Nevertheless, Foucault, Courvalin and Grillot-Courvalin found that VanA-type resistance in MRSA, although energetically expensive when induced, is only minimal in biological cost in the absence of induction. Therefore, the continued threat of increased dissemination and frequency of VRSA infections should not be discounted [468]. 

The global prevalence of VRSA has been increasing steadily over the past two decades [398,466]; 2% before 2006, 5% between 2006–2014 and 7% between 2015–2020 for a 3.5-fold increase between pre-2006 and 2020. The rate of VRSA among *S. aureus* isolates was 16% in Africa, 5% in Asia, 4% in North America, 3% in South America and 1% in Europe [398]. Infection rates of VRSA have been shown to mirror those of VRE and VISA. Higher burdens of VRSA disease in lower- and middle-income continents of Africa and Asia have been attributed to poorer hygiene, reduced implementation of antimicrobial stewardship and limitations in epidemiological surveillance [279,280,281,282,283,284,285,398]. There have been no reports of VRSA in Oceania [398,466]. 

Like enterococci, *S. aureus* are major causative agents of disease in livestock. Exemplified by diseases such as mastitis in goats and cattle, as well as “bumblefoot” in chickens [297], *S. aureus* outbreaks frequently result in significant economic losses [298]. Although VISA and VRSA strains have been isolated from livestock [471,472,473], reports of their incidence in the literature are rare compared to other vancomycin-sensitive strains such as MRSA [474,475]. One explanation for this is the comparatively higher fitness cost of VISA and VRSA lineages [463,468]. The global distribution of VRSA and VISA in agriculture is unknown, but presumably highest in developing nations due to their high quantity of antimicrobial consumption, high prevalence of intensive farming and lower levels of hygiene, antimicrobial stewardship and surveillance [279,280,281,282,283,284,285,289,398,476,477].

## 3. Alternative Treatment Options for Vancomycin Resistant Infections

Treatment options for VRE, VISA and VRSA are limited to several antibiotic classes. Clinically, linezolid is employed to treat VISA [478], VRSA and VRE [479]; tigecycline against VRE [480,481] and daptomycin against VRE and VRSA [355,482,483] (note: daptomycin is not FDA-approved for VRE, but has been used off-label against VRE infection [484]). Unfortunately, resistance to each of these antibiotics has emerged. As such, the use of modified glycopeptide derivatives such as dalbavancin, oritavancin, and telavancin, and/or combinational antibiotic therapy is typically exercised as a way to overcome AMR [485]. While there remains a limited supply of novel antibiotics in development [486,487,488,489], the emergence of resistance to future approved antibiotic treatment regimens is expected [490]. Although R&D pathways surrounding new-class antibiotics represent a possible path forward, these programs remain high risk, expensive, and time-consuming endeavors that many pharmaceutical companies have withdrawn from [9,491,492,493]. [226] Therefore, alternative approaches to treat drug-resistant *Enterococcus* and *S. aureus* infections are needed which may substitute/complement existing antibiotic therapy. 

### 3.1. Antibiotic-Chemoattractant Conjugants

Antibiotic-chemoattractants consist of a formylated peptide (neutrophil chemoattractant) covalently linked to vancomycin. Vancomycin’s selective binding to the bacterial cell wall allows for targeted recruitment of neutrophils directly to the site of infection. Enhanced neutrophil recruitment, phagocytosis and killing of *S. aureus* was observed *in vitro* and mice *in vivo* in addition to potentiation of neutrophil activity through optimization of the formyl peptide sequence [494]. Vancomycin-lipopeptide conjugates with high antibacterial activity against VRE *in vitro* and cytocompatibility in Wistar rats *in vivo* have also been reported [495]. 

### 3.2. Antibody-Antibiotic Conjugants

Antibody-antibiotic conjugants (AACs) consist of an antibiotic payload linked to a pathogen-specific antibody for targeted delivery. AACs have been used successfully to clear intracellular *S. aureus* reservoirs in mice [496,497] where the bacteria are normally protected from conventional antibiotics which are poor at intracellular penetration and mostly inactive against dormant bacteria [497]. Conjugants can be optimally customized to the pathogen through use of alternative delivery systems (e.g., nanoparticles) and payloads (e.g., different antibiotics/antibacterial compounds) that increases target specificity, absorption and reduces off-site toxicity as appropriate [498,499]. 

As a proof of concept, antibody-conjugated nanocarrier-delivered rifampicin demonstrated superior antibacterial activity against *S. aureus* biofilms *in vitro* and in a mouse infection model compared to rifampicin in free form [499]. Rifamycin-class antibiotics covalently linked to the anti-*S. aureus* antibody THIOMAB were also superior to vancomycin in a murine of MRSA bacteremia [496]. In another study, a THIOMAB AAC, either as a monotherapy or in combination with vancomycin, demonstrated a more sustained and superior antibacterial activity in mice compared to vancomycin alone [500]; THIOMAB AAC also displayed favorable pharmacokinetic profiles in rats and monkeys [501,502]. In 2020, DSTA4637S, a THIOMAB AAC completed phase 1b clinical trials to treat *S. aureus* bacteremia [503,504]. Attempts at engineering antibody-antibiotic conjugants against VRE have not been reported in the literature.

### 3.3. Antimicrobial Peptides and Polymers

Antimicrobial peptides and polymers are natural and synthetic [505,506] compounds with broad-spectrum antibacterial activity [507]. Antimicrobial peptides are small (10–50 amino acids), amphiphilic and cationic molecules which facilitates their accumulation and formation of cytocidal pores on bacterial cell membranes [508]. Antimicrobial peptides with rapid bactericidal activity against multidrug-resistant organisms, including *S. aureus* and enterococci, have been reported in the literature [509,510,511]. 

For example, mesenchymal stem cells (MSC) possess direct antibacterial activity through the secretion of antimicrobial peptides. Against *S. aureus*, Yagi et al. showed that adipose-derived human MSC conditioned media significantly inhibited *S. aureus* growth *in vitro* even without continued adipose-derived human MSC presence, and antimicrobial peptide production, namely LL-37, could be enhanced with the addition of vitamin D. *In vivo*, Johnson, Webb and Dow demonstrated that MSC therapy also induced antimicrobial peptide production and enhanced antibiotic treatment against a chronic *S. aureus* biofilm infection in mice [512,513,514]. Non-human antimicrobial peptides, such as MPX from wasp venom have also demonstrated efficacy against *S. aureus in vitro* and in a mouse wound infection model [515]. The currently known antimicrobial peptides that have exclusively shown antibacterial activity against VISA and VRSA, and their possible mechanisms of action have been reviewed by Hernández-Aristizábal and Ocampo-Ibáñez [516]. 

Antimicrobial peptides active against *Enterococcus* include C16-KGGK [517], KP, L18R [518], buwchitin [519], Bip-P-113 [520], FK13-a1, FK13-a7 [521], AMP2 [522], WLBU2, LL-37 [523], SAAP-148 [524] and H4 [525]. However, as clinical trials show that most antimicrobial peptides are only effective topically [526], their use as direct antimicrobials may be limited to treating superficial wound infections only.

Antimicrobial peptides can be further modified into antimicrobial polymers [509] and designed based on their intended target pathogen(s) and desired sites/modes of action [507,527,528]. Due to their multimodal mechanisms of activity, antimicrobial peptides and polymers can also prevent bacterial resistance development [509,529]. As such, both antimicrobial peptides and polymers have a broad range of possible clinical applications from acting as direct antimicrobials, as an antibiotic synergist or maintaining sterility on medical device surfaces [509,526,530,531]. Antimicrobial polymers active against *S. aureus* include peptoid polymers [532], NP108 [533] and ammonium ethyl methacrylate homopolymers [534] while photo-antimicrobial polymers based on Rose Bengal (a singlet oxygen photosensitiser) and cationic polystyrene have demonstrated activity against both *S. aureus* and *E. faecalis* [535]. Antimicrobial peptides may also be formulated with antimicrobial polymers for enhanced efficacy; the peptide-synthetic polymer conjugate which consisted of the antimicrobial peptide C16-KGGK formulated with a biodegradable polymer exhibited strong and improved anti-*E. faecalis* activity compared to C16-KGGK alone [517].

### 3.4. Bacteriophage Therapy

AB-SA01 is a cocktail of three obligately lytic *Staphylococcus* myoviruses that killed 95% of 205 multidrug-resistant clinical *S. aureus* isolates *in vitro* including methicillin-resistant and vancomycin-intermediate strains. Resistance emergence was scarce (≤3 × 10^−9^), and bacterial resistance to one phage component could be abrogated by the activities of other component phages. In mice, AB-SA01 reduced lung *S. aureus* populations on par with vancomycin treatment and no adverse reactions were reported in human subjects upon administration [536,537]. AB-SA01 was demonstrated to be safe and well tolerated in two separate phase I clinical trials [536,538]. Other phages tested for anti-staphylococcal efficacy include PYO^Sa^ [539], JD419 [540], vB_SauH_2002 and phage 66 [541]. A recent 2022 review of the bacteriophage animal models, treatments and clinical trials used to treat *S. aureus* infections has been published by Plumet et al. [542]. 

Enterococcal bacteriophages include MDA1 and MDA2 [543], VPE25, VFW [544], phi phages [545], vB_EfaS-Zip, vB_EfaP-Max [546], vB_EfaS_HEf13 [547], vB_EfaS_efap05-1 [548], EF-P29 [549], Efv12-phi1, EFLK1, Ef11, EF-P10, PlyV12 [550], SSsP-1, GVEsP-1 [551], EFDG1 [552] and vB_EfaM-LG1 [553]. Currently, studies using lytic phages against *Enterococcus* are limited to *in vitro* and animal models only, with no clinical or single-arm trials conducted in recent times [550]. However, case reports detailing the clinical successes of phage therapy against *E. faecalis* [554,555] and *E. faecium* [556] infections suggests that bacteriophage therapy remains a viable alternative to antibiotics in the fight against AMR pathogens.

### 3.5. Centyrins

Centyrins are small globular proteins derived from the fibronectin type III-binding domains of human tenascin-C proteins. These biologic compounds are able to bind to *S. aureus* leukocidins with high affinity, preventing the destruction of human immune cells from toxin-mediated cytolysis. Centyrins proved effective in an *in vivo* model of murine intoxication as well as murine models of prophylactic and therapeutic treatments of systemic *S. aureus* infections [557]. Currently, there are no reported studies of centyrins against enterococcal infections in the literature. These results demonstrate the therapeutic potential of antimicrobials that focus on neutralising bacterial virulence factors in contrast to traditional therapies that primarily directly target the bacteria. 

### 3.6. Clustered Regularly-Interspaced Short Palindromic Repeats (CRISPR) and CRISPR-Associated Genes (CRISPR/Cas)

CRISPR is a prokaryotic self-defense mechanism that is inversely correlated to the acquisition of antibiotic resistance in *E. faecalis* and *E. faecium*, suggesting that antibiotic selection indirectly selects against *Enterococcus* genomic integrity through loss of CRISPR. This is reinforced by *in vivo* experiments that demonstrated CRISPR-mediated inhibition of plasmid dissemination. In addition, maintenance of self-targeting, chromosome cleaving CRISPR in *E. faecalis* appeared to come at a fitness cost. Given that functional CRISPR systems are observed to be absent in multidrug-resistant *E. faecalis* [558], selective introduction and maintenance of CRISPR in virulent bacteria via physical (e.g., microinjection), biological (liposomes) or viral (e.g., adenoviruses) vectors [559] may be a viable method of selecting against horizontal gene transfer and fitness as a means of combatting antibiotic-resistant infections [558,560,561,562]. 

CRISPR/Cas is also present in clinical MRSA [563], and can be artificially engineered for genome editing in *S. aureus* through downregulation, mutation, insertion and/or deletion of key genes [564,565,566,567,568]. More, CRISPR/Cas can be exploited to specifically target virulent *S. aureus* sub-populations [569,570], destroy AMR-carrying MGEs, and “immunise” avirulent staphylococci to prevent uptake of resistance-conferring MGEs. The selective recognition of virulence genes in *S. aureus* by CRISPR/Cas may also serve as a useful tool in the detection and differentiation of clinical *S. aureus* strains [569,571].

### 3.7. Direct Lytic Agents (DLAs) 

DLAs are novel antimicrobials that act through swift destabilization of bacterial cell walls and bacteriolysis, with the intended aim to synergise with and complement existing antibiotics without causing resistance development against DLAs. They encompass two classes of purified polypeptides—lysins (peptidoglycan hydrolase enzymes) and amurins (targeting outer membrane peptides). They are active against Gram-positive bacteria, with one of them, exebacase, having entered into Phase III clinical trials [572] against *S. aureus* bacteremia and right-sided endocarditis [573]—the first and only agent of the lysin-class to do so [572,574]. Lysins can also be secreted by bacteriophages as enzymes [575]. When administered intranasally, the anti-staphylococcal lysin SAL-200 protected mice from lethal *S. aureus* pneumonia [576] and progressed into a phase 2a clinical trial [577]. Another lysin, PlySs2, was protective against mixed MRSA and *Streptococcus pyogenes* bacteremia infection in mice with no bacterial resistance observed [578].

Lysins active against enterococci have also been reported [579], and lysins can be engineered to be active against both staphylococci and enterococci [580]. Both lysins and amurins are currently undergoing commercial development as therapeutics against AMR bacteria [581]. 

### 3.8. Fecal Microbiota Transplantation (FMT)/Probiotic Intervention

FMT describes the restoration process of a recipient’s gut microbiota to a normal composition through fecal transplantation from a healthy donor [582]. FMT and the probiotic *Lactobacillus* rapidly reduced gut VRE colonization and restored microbiota diversity in mice compared to control groups. Clinically, FMT has successfully helped cure patients of MRSA enteritis and restore microbiota balance [583], as well as decolonize VRE without adverse effects [584,585,586].

### 3.9. Drug Repurposing

The anti-inflammatory drug ebselen and the anticancer drugs adarotene, floxuridine and streptozotocin showed antibacterial activity against VISA and VRSA. Oral ebselen increased mice survival by 60% in a lethal septicemic MRSA model compared to control. Adarotene protected *Caenorhabditis elegans* from MRSA-induced death, while floxuridine protected human neutrophils from S. aureus killing, inhibited S. aureus growth and virulence regulation, and may cause bacterial DNA damage in a murine blood infection model. Streptozotocin displayed similar anti-staphylococcal efficacy compared to floxuridine, but was less effective at protecting neutrophils and did not inhibit the growth of S. aureus. Neither floxuridine nor streptozotocin showed noticeable side effects of abnormal blood cell counts or glucose levels at the experimental concentrations used in mice [587,588,589]. 

Against E. faecium and E. faecalis, the clinically approved antihelminthic drug bithionol showed significant antibacterial and antibiofilm effects in a dose-dependent manner in vitro and remarkably reduced bacterial burdens in mouse organs in combination with antibiotics in a peritonitis infection model [590]. Auranofin, a FDA-approved drug for rheumatoid arthritis, also demonstrated potent in vitro efficacy against enterococci, no resistance development over 14 passages, antibiofilm effects and superior in vivo activity in mice when compared to the clinical VRE antibiotic linezolid [591]. Given that drug repurposing focuses on using approved existing drugs, this concept holds promise for reduced time and cost of development, as well as swifter clinical trials than drug development de novo [592].

### 3.10. Host-Directed Therapy (HDT)

HDT focuses on the manipulation of host factors to the detriment of the pathogen in infection. This may be achieved through blocking host proteins or pathways required for pathogenesis and stimulation/reduction of immune responses as appropriate. Zhu et al. showed that use of the autophagy inhibitor 3-MA reduced MRSA autophagy by macrophages, reduced MRSA population size and potentiated macrophage phagocytosis of MRSA. Similar positive outcomes were reproducible an *in vivo* mouse model [593,594]. Clinically, HDT research can be applied to combat diseases such as septicemia through modulation of cytokine activity [593], a concept which has seen positive outcomes in clinical trials [595,596]. 

### 3.11. Nanoparticles

Tan et al., showed that lipid-polymer hybrid nanoparticles (LPNs) could effectively load and deliver ampicillin (Amp) to *E. faecalis* and its biofilm in a protozoa infection model. Protozoa receiving Amp-LPNs exhibited significantly reduced populations of *E. faecium* compared to free ampicillin treatment groups in simulated infection models of prophylaxis, acute and chronic infections. LPNs greatly potentiated ampicillin activity at late interventions, and Amp-LPNs boosted the survival of protozoa by almost 400% at 40 h post infection, with no viable protozoa remaining in any pure ampicillin treatment groups [597]. In the root canals of beagle dogs, nano-scale silver-zinc-calcium-silica particles showed strong preventative effects against *E. faecalis* infection [598]. Nanosilver has also shown antibacterial efficacy against *S. aureus* [599], and rifampicin-loaded nanoparticles were successful in treating MRSA at a reduced antibiotic dosage compared to free drug in a mice wound infection model [600].

### 3.12. Reversing Antibiotic Resistance

PBT2 is a safe-for-human-use zinc ionophore previously developed to treat neurodegenerative diseases, which has been shown to break resistance to multiple classes of antibiotics in a variety of animal models [601,602,603,604]. Bohlmann et al. showed that PBT2, in the presence of zinc, is bactericidal against MRSA and VRE. In addition, it also reverses acquired bacterial resistance to many clinical antibiotics including vancomycin at sub-inhibitory concentrations. The combination of PBT2 + zinc + vancomycin also significantly reduced VRE infection in a murine wound infection model. The authors were unable to select for mutants resistant to PBT2 + zinc treatment [601]. PBT2 and zinc was also able to break intrinsic polymyxin resistance in MRSA and VRE *in vitro* as demonstrated by De Oliveira et al. [603], highlighting the utility of PBT2 to reverse both intrinsic and acquired mechanisms of antibiotic resistance in bacteria. Other studies investigating the utility of using natural products [605], traditional medicines [606] and other existing non-antibiotic drugs [607,608] in reversing antibiotic resistance in *S. aureus* and/or enterococci have also been published.

### 3.13. Vaccination

Nontoxigenic protein A (SpA(KKAA)) is a mouse immunogen and stimulates humoral immune responses against the *S. aureus* surface protein staphylococcal protein A (SpA). SpA is a B cell superantigen that promotes B cell apoptosis and interferes with opsonophagocytosis. Kim et al. showed that vaccination of mice with monoclonal antibodies to SpA(KKAA) neutralized the ability of SpA to inhibit opsonophagocytosis, attenuated *S. aureus* virulence and potentiated antibacterial immunity. In another mouse model, Chen et al. demonstrated that systemic administration of a recombinant neutralizing antibody for SpA can promote IgA and IgG responses in addition to decolonization of *S. aureus* [609,610,611]. A SpA-targetting monoclonal antibody, 514G3, was safe and well tolerated in a phase I clinical trial [612] and a phase II clinical trial followed [613] with SpA vaccine optimisation efforts in progress [614]. Clinical trials involving other *S. aureus* vaccine candidates are ongoing [615,616], but no vaccines are currently approved for clinical use [616,617].

Enterococcal proteins and polysaccharides have been the targets of potential vaccine candidates. Antisera raised to enterococcal polysaccharides have been shown to promote opsonic killing *in vitro* and protect against enterococcal bacteremia *in vivo*, while vaccination with recombinant enterococcal virulence factors and antigens also proved to be opsonic *in vitro* and promoted bacterial clearance in mice [618]. Multi-epitope vaccines [619] and conjugated vaccines [620,621] have also demonstrated promising results. However, there are currently no *Enterococcus* vaccines in development, and none have been approved for clinical use [616,619].

## 4. Conclusions

*E. faecalis*, *E. faecium* and *S. aureus* are common human commensal organisms with potential to cause serious, life-threatening infections with exceptional intrinsic and acquired antibiotic resistance capabilities that have increased in prevalence globally in recent decades. This is enabled by the emergence and continued dissemination of mobile *van* resistance cassettes amongst staphylococci and enterococci through MGEs, which confer high-level vancomycin resistance through modification of the D-Ala-D-Ala peptidoglycan terminal ends in addition to endogenous, non-transferable mutations that give rise to intermediate-level resistance in *S. aureus*. Although other viable antibiotic combinations are still available for vancomycin resistant infections, novel antibiotics, in addition to alternative non-drug antimicrobial strategies will likely be needed to ensure treatment options remain for increasingly drug-resistant infections in the future. 

## Figures and Tables

**Table 3:** The molecular basis of *van*-mediated vancomycin resistance in enterococci.

Gene	Protein/Function	Mechanism of Action	References
D-Ala-D-Lac based resistance (VanA-type resistance)—*vanA*, *vanB*, *vanD*, *vanF*, *vanM* gene cassettes High level vancomycin resistance			
*vanA* ^1^	Ligase	Catalyses the formation of D-Ala-D-Lac depsipeptides	[192,373]
*vanH*	Dehydrogenase	Catalyses conversion of pyruvate to D-lactate, generating the necessary substrate for D-Ala-D-Lac depsipeptide synthesis	[370,374]
*vanR/vanS*	Regulatory System	The vanR transcription regulator and the vanS sensor kinase comprise the canonical two-component regulatory system that controls *vanHAX* expression	[375]
*vanX*	Dipeptidase	Cleavage of D-Ala-D-Ala into individual D-Ala residues, thus depleting D-Ala-D-Ala dipeptide substrates from the peptidoglycan synthesis pathway; inhibition of D-Ala-D-Ala synthesis and subsequent loss of binding sites for vancomycin	[376]
*vanY*	Pentapeptidase	D,D-carboxypeptidase activity against D-Ala; reducing availability of D-Ala precursors and therefore favoring the production of peptidoglycan with D-Ala-D-Lac terminals	[377,378,379,380,381]
*vanZ*	Unknown	Currently unknown; *vanZ* does not appear to be necessary for vancomycin resistance but is required for resistance to the related glycopeptide teicoplanin	[382,383]
**D-Ala-D-Ser based resistance (VanC-type resistance)—*vanC ^2^*, *vanE*, *vanG*, *vanL*, *vanN* gene cassettes Low level vancomycin resistance**			
*vanC* ^1^	Ligase	Synthesis of D-Ala-D-Ser peptidoglycan terminals	[371]
*vanR*/*vanS*	Regulatory system	Two-component regulatory system consisting of the VanR transcription regulator and the VanS sensor kinase	[371]
*vanT* ^3^	Membrane-bound serine racemase	Catalyses conversion of L-Ser to D-Ser, producing the D-Ser substrates required for D-Ala-D-Ser terminals	[384,385,386,387,388,389,390,391,392,393,394]
*vanXY* ^3^	Bifunctional dipeptidase/pentapeptidase	Hydrolyses UDP-MurNac-pentapeptides (D-Ala) and D-Ala-D-Ala	[395,396]

^1^*vanA* and *vanC* are the ligase genes of the *vanA* and *vanC* operons respectively. Ligase genes are named similarly for the other resistance cassettes e.g., *vanB* is the ligase gene designation for the *vanB* operon [371]. ^2^ The *vanC* operon is not found in *E. faecium* or *E. faecalis* [397]. ^3^ Not found in D-Ala-D-Lac based vancomycin resistance cassettes [371].

## Data Availability

Not applicable.
